# Development and External Validation of an Interpretable Machine Learning Framework for Predicting Pneumothorax-Associated Acute Kidney Injury: A Multicenter Retrospective Study

**DOI:** 10.3390/jcm15145599

**Published:** 2026-07-16

**Authors:** Guanghao Pan, Jingli Fan, Wenhao Wang, Guang Yang, Jianling Su, Huining Liu

**Affiliations:** 1 Department of Thoracic Surgery, First Hospital of Hebei Medical University, Shijiazhuang 050031, China; 25034100949@stu.hebmu.edu.cn (G.P.); fanjingli2025@163.com (J.F.); wangwenhao2026@163.com (W.W.); yangguangsurgeon@163.com (G.Y.); 2 Department of Critical Care Medicine IV, First Hospital of Hebei Medical University, Shijiazhuang 050031, China

**Keywords:** pneumothorax, acute kidney injury, machine learning, intensive care unit, predictive model, restricted cubic spline, explainable artificial intelligence, nomogram

## Abstract

**Background/Objectives:** Intensive Care Unit (ICU) patients with pneumothorax face an elevated risk of developing Acute Kidney Injury (AKI) due to compromised hemodynamics and increased intrathoracic pressure. Early identification is crucial but challenging. This study aimed to develop and externally validate an interpretable machine learning (ML) framework to predict pneumothorax-associated AKI. **Methods:** This multicenter retrospective study utilized data from the MIMIC-IV database (development), alongside a temporal validation cohort (MIMIC-III) and an independent external validation cohort (eICU). A tri-algorithm intersection strategy—comprising Boruta, Least Absolute Shrinkage and Selection Operator (LASSO), and Recursive Feature Elimination (RFE)—was applied to extract optimal predictors. We systematically evaluated nine supervised ML algorithms. Shapley Additive exPlanations (SHAP) and Restricted Cubic Spline (RCS) analyses were integrated to unveil decision-making mechanics and non-linear dynamics. The optimal model was deployed as a web-based dynamic nomogram. **Results:** The hybrid feature selection strategy identified a parsimonious consensus of 7 core predictors (BUN, SOFA score, CKD, PEEP, Heart Failure, Albumin, and Age). Following comprehensive evaluation, the Logistic Regression model demonstrated favorable discriminative performance [Area Under the Curve (AUC) = 0.839 (95% CI: 0.786–0.891), Sensitivity = 81.5%, Specificity = 76.0%] and external generalizability (eICU AUC = 0.869; MIMIC-III AUC = 0.854). SHAP analysis delineated the individual contribution of each feature. RCS analysis revealed significant non-linear, dose–response relationships, highlighting an exponential AKI risk escalation driven by elevated BUN and higher PEEP levels. Decision Curve Analysis (DCA) suggested the potential net clinical benefit of the model across all validation cohorts. **Conclusions:** We developed a transparent, externally validated ML framework for predicting pneumothorax-associated AKI. The resulting web-based nomogram provides intensive care physicians with a practical, data-driven bedside tool to assist in personalized risk stratification. However, given the substantial calibration drift observed in the external cohort, local recalibration is essential prior to its use outside the MIMIC-derived population, and prospective cohort validation remains necessary before routine clinical implementation.

## 1. Introduction

Pneumothorax is a critical and potentially life-threatening respiratory emergency frequently encountered in the Intensive Care Unit (ICU) [1]. Beyond the immediate compromise of pulmonary gas exchange, severe pneumothorax significantly elevates intrathoracic pressure [2]. This mechanical shift directly impedes systemic venous return and impairs cardiac output, initiating a cascade of severe hemodynamic instability [2,3]. Consequently, organs sensitive to perfusion pressure, particularly the kidneys, are vulnerable. Acute Kidney Injury (AKI) has emerged as a frequent and severe complication in critically ill patients with pneumothorax, strongly linked to prolonged mechanical ventilation, increased healthcare costs, and significantly higher short-term mortality [4,5]. Early identification of patients at high risk of developing pneumothorax-associated AKI is therefore of great clinical importance.

Traditionally, intensive care physicians have relied on generalized prognostic scoring systems, such as the Sequential Organ Failure Assessment (SOFA) score, to stratify illness severity [6]. However, these tools are fundamentally reactive rather than proactively predictive, and they often fail to capture the specific mechanistic cascades—such as the interplay between positive end-expiratory pressure (PEEP) loads and renal hypoperfusion—unique to pneumothorax patients [6,7]. Standard linear statistical models struggle to accurately delineate the complex, non-linear dose–response relationships inherent in critical illness trajectories [8].

In recent years, Machine Learning (ML) has significantly advanced clinical predictive modeling by excelling at identifying multidimensional, non-linear patterns within large-scale Electronic Health Records (EHRs) [9]. Despite achieving discriminative performance, the widespread clinical adoption of advanced ML algorithms (e.g., ensemble trees or deep neural networks) has been severely hindered by their inherent “black-box” nature [10]. In high-stakes ICU settings, clinicians require not only accurate probability scores but also transparent, pathophysiologically sound explanations for algorithmic decisions—a paradigm recognized as Explainable Artificial Intelligence (XAI) [11,12].

To address these critical methodological and clinical gaps, this multicenter retrospective study leveraged high-resolution data from the MIMIC-IV, MIMIC-III, and eICU Collaborative Research databases. The primary aim of this study was to develop and externally validate an interpretable ML framework for early risk prediction of pneumothorax-associated AKI. By utilizing a rigorous tri-algorithm feature selection strategy (Boruta, LASSO, and RFE) and systematically comparing nine ML algorithms, we sought to identify the optimal predictive model. By integrating Shapley Additive exPlanations (SHAP) and Restricted Cubic Spline (RCS) analyses, we aimed to demystify the algorithmic decision-making process and unveil crucial non-linear clinical dynamics. Ultimately, the best-performing model was deployed as a dynamic web-based nomogram, demonstrating that a parsimonious, transparent algorithm can provide a net clinical benefit for personalized bedside triage.

## 2. Materials and Methods

### 2.1. Study Design and Ethical Considerations

This retrospective study utilized clinical information from three independent, publicly accessible critical care databases. The Medical Information Mart for Intensive Care IV (MIMIC-IV, version 2.2) served as the primary cohort for model development and internal validation [13]. To ensure the spatial and temporal robustness of our predictive framework, a temporal validation cohort (MIMIC-III, version 1.4) [14] and an independent external validation cohort (eICU-CRD) [15] were established. Our reporting protocol complied with the Transparent Reporting of a multivariable prediction model for Individual Prognosis Or Diagnosis (TRIPOD) statement [16] and the recent TRIPOD + AI statement for reporting clinical prediction models that use machine learning methods [17]. Given the anonymized nature of the health records in these databases, the institutional review boards associated with PhysioNet granted a waiver for formal ethical review and individual informed consent. Finally, data extraction was performed only after the investigating team completed the mandatory Collaborative Institutional Training Initiative (CITI) coursework and obtained approved credentialing from PhysioNet.

### 2.2. Study Population and Data

Preprocessing Adult patients (age ≥ 18 years) diagnosed with pneumothorax upon ICU admission were screened. To ensure baseline homogeneity, exclusion criteria included: (1) non-first ICU admission; (2) ICU stay < 24 h; (3) HIV infection; (4) pregnancy; (5) severe immune-compromising hematological malignancies (leukemia, lymphoma, multiple myeloma). The final MIMIC-IV cohort (*n* = 1040) served as the development set, which was randomly partitioned into a training set (70%) and an internal validation set (30%). A temporal validation cohort and an independent external validation cohort were extracted from the MIMIC-III and eICU databases, respectively. To prevent data leakage due to the overlapping timeframe (2008–2012) and patient populations between MIMIC-III and MIMIC-IV, a deduplication procedure was applied; patients in the MIMIC-III database admitted during the overlapping years were systematically excluded to ensure mutual exclusivity between the internal development and external temporal validation cohorts. Clinical features within the first 24 h of admission were extracted. Variables with >20% missing values were excluded. To minimize bias, the remaining missing data were addressed using the Multivariate Imputation by Chained Equations (MICE) algorithm via the ‘mice’ package in R. Specifically, we generated 5 imputed datasets using predictive mean matching for continuous variables and logistic regression for categorical variables. The imputation model included all candidate predictors alongside the primary outcome variable (AKI) to preserve the underlying data structure. Finally, the analytical results were pooled according to Rubin’s rules. The primary outcome of this study was the occurrence of AKI during the ICU stay. AKI was defined according to the Kidney Disease: Improving Global Outcomes (KDIGO) 2012 clinical practice guidelines [18]. Specifically, AKI was identified by either a serum creatinine increase of ≥0.3 mg/dL (≥26.5 µmol/L) within 48 h, a serum creatinine rise to ≥1.5 times baseline within the prior 7 days, or a urine output of <0.5 mL/kg/h for 6 h. Baseline creatinine was defined as the lowest creatinine value recorded within the 7 days prior to ICU admission. For dynamic laboratory variables and clinical parameters extracted within the first 24 h of ICU admission, both the maximum and minimum values were evaluated. To capture the most severe disease state, the value representing the worst clinical physiological condition (e.g., the maximum value for BUN and the minimum value for Albumin) was selected for final modeling.

### 2.3. Optimal Feature Selection Strategy

To overcome the inherent biases and overfitting risks associated with single-algorithm selection, a tri-algorithm intersection strategy was utilized. First, the Boruta algorithm was applied for holistic feature evaluation. As a random forest-based wrapper method, Boruta comprehensively captures all relevant attributes by iteratively comparing the importance of original features against randomized “shadow features” [19]. Second, Least Absolute Shrinkage and Selection Operator (LASSO) regression was employed. By introducing an L1 regularization penalty, LASSO enforces model sparsity; optimized via 10-fold cross-validation, it reduces multicollinearity in high-dimensional data [20]. Finally, Recursive Feature Elimination (RFE) was implemented. Operating as a greedy optimization algorithm, RFE systematically ranks feature importance by iteratively building models and discarding the weakest variables to determine the optimal feature subset for maximal predictive performance [21]. Ultimately, to ensure reliability and generalizability, only the independent variables uniformly identified by the strict intersection of these three distinct mathematical algorithms were retained to formulate the parsimonious optimal subset. The intersection strategy of Boruta, LASSO, and RFE was deliberately employed to construct a parsimonious model, retaining consistent predictors to minimize dataset-specific noise and prevent overfitting in high-dimensional critical care settings.

### 2.4. Model Development and Comprehensive

To explore the clinical landscape and multicollinearity of the optimal feature subset, a Spearman correlation matrix was constructed. Additionally, an unsupervised hierarchical clustering heatmap was generated to visualize the standardized expression patterns (Z-scores) of these features across AKI and non-AKI cohorts.

Nine supervised machine learning algorithms were developed: Logistic Regression (LR), Support Vector Machine (SVM), Gradient Boosting Machine (GBM), Neural Network (NN), K-Nearest Neighbors (KNN), XGBoost, AdaBoost, LightGBM, and CatBoost. To mitigate the bias introduced by imbalanced binary AKI outcomes, we assigned adjusted class weights to positive AKI samples during model training for all nine machine learning algorithms. Combined with separated multiple imputation and independent feature selection between training and validation cohorts, this dual strategy minimizes potential data leakage and reduces classification bias caused by uneven event rates. Models were optimized via grid search and 10-fold cross-validation. Model discrimination was evaluated using the Area Under the Receiver Operating Characteristic Curve (AUC), and pairwise statistical comparisons across all nine machine learning algorithms were conducted via DeLong’s test to objectively quantify performance differences. All corresponding Z statistics and *p*-values from pairwise AUC comparisons are summarized in Supplementary Table S2. Statistical comparisons demonstrated that the logistic regression model exhibited non-inferior discriminative capacity relative to complex black-box algorithms (XGBoost, CatBoost, GBM, SVM, Neural Network, all *p* > 0.05), while it significantly outperformed KNN, AdaBoost and LightGBM (all *p* < 0.05). Calibration was assessed via Brier scores and calibration curves, while clinical utility was quantified using Decision Curve Analysis (DCA). (The detailed mathematical formulation and specific predictive equation for the model are provided in Appendix A).

To evaluate predictive accuracy, confusion matrices were generated across all validation cohorts to evaluate the true positive, true negative, false positive, and false negative rates. To ensure biological plausibility across different mathematical architectures, the feature importance scores from all nine algorithms were extracted, normalized, and visually compared. To validate the robustness of the optimal model, subgroup analyses were performed to calculate AUCs across distinct clinical strata, including different age groups, comorbidities, and PEEP exposure levels.

### 2.5. Model Interpretation and Clinical

Translation Shapley Additive exPlanations (SHAP) were implemented to elucidate the “black-box” decision-making mechanics, quantifying global feature importance, directional impacts, and individual patient-level local predictions. A multivariable logistic regression model was utilized to calculate Odds Ratios (ORs) and 95% confidence intervals (CIs), visualized using a forest plot. Concurrently, Restricted Cubic Spline (RCS) analysis was performed to uncover and validate the non-linear dose–response relationships between continuous clinical variables and AKI risk.

For direct bedside clinical translation, a static density-enhanced nomogram was constructed to provide a visual scoring system. Additionally, an interactive dynamic web-based calculator was developed using the Shiny and DynNom frameworks to enable real-time, precise risk computation.

### 2.6. Statistical Analysis and Generative

AI Disclosure All statistical analyses were performed using R software (version 4.2.1). A two-sided *p*-value < 0.05 was considered significant. During the preparation of this manuscript, generative artificial intelligence tools were utilized strictly for superficial English language editing and readability enhancement after the original draft was completed by the authors. No AI tools were used in study design, data extraction, statistical modeling, or clinical interpretation.

## 3. Results

### 3.1. Baseline Characteristics of the Study Population

**Figure 1:**
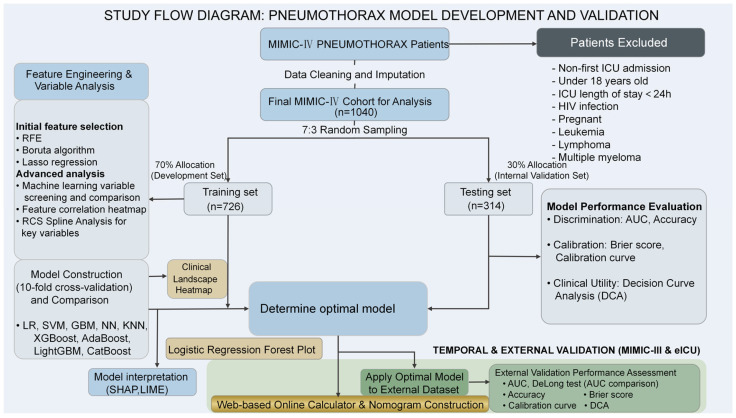
Study flow diagram outlining the comprehensive development and validation of the machine learning predictive framework for pneumothorax- associated Acute Kidney Injury (AKI). The process encompasses initial cohort definition and stringent exclusion criteria from the MIMIC-IV database, yielding a final cohort of 1040 patients. This cohort was randomly partitioned into a training set (70%) and an internal testing set (30%). The methodology features a rigorous feature engineering pipeline utilizing a tri-algorithm intersection (RFE, Boruta, LASSO), followed by the systematic evaluation of nine diverse machine learning algorithms via 10-fold cross-validation. The optimal model’s generalizability was subsequently evaluated using a temporal validation cohort (MIMIC-III) and an independent external validation cohort (eICU). Finally, the framework was interpreted using explainable artificial intelligence (SHAP/LIME) and successfully deployed as both a static nomogram and a web-based online calculator for real-time clinical application.

A total of 1040 eligible adult patients diagnosed with pneumothorax upon ICU admission from the MIMIC-IV database were enrolled in this study as the development cohort. As illustrated in the study flow diagram (Figure 1), this primary population was screened based on predefined inclusion and exclusion criteria. To facilitate model construction and internal validation, the final analysis cohort was randomly partitioned in a 7:3 sampling ratio, yielding a training set of 726 patients and an internal validation testing set of 314 patients. To evaluate the geographical and clinical generalizability of our predictive framework, a temporal validation cohort and an independent external validation cohort were established using high-resolution clinical data extracted from the MIMIC-III and eICU databases.

The core baseline demographic, clinical, and laboratory characteristics of the development population, stratified by the subsequent development of ICU-acquired Acute Kidney Injury (AKI), are summarized in Table 1. A comprehensive baseline table encompassing all evaluated clinical variables, including those without statistical significance, is provided in Supplementary Table S1. Within the primary cohort, 338 patients (32.5%) developed AKI during their ICU stay. Among the 338 patients who developed AKI, the specific KDIGO staging distribution could not be reliably determined due to the limitations of the extracted clinical documentation within the database, which primarily captured the occurrence of AKI rather than granular staging metrics. Univariate statistical analysis revealed significant physiological and therapeutic disparities between the non-AKI and AKI groups. Patients who progressed to AKI were significantly older [median 67 (IQR: 57–79) vs. 62 (43–71) years, *p* < 0.001] and exhibited a significantly higher baseline prevalence of chronic organ dysfunctions, including Chronic Kidney Disease (CKD) (21.6% vs. 4.3%, *p* < 0.001), Heart Failure (34.9% vs. 13.4%, *p* < 0.001), and COPD (21.3% vs. 14.2%, *p* = 0.005).

Regarding laboratory parameters, the AKI cohort demonstrated systemic and metabolic derangements upon ICU admission, characterized by substantially elevated Blood Urea Nitrogen (BUN) [median 26.5 vs. 15.0 mg/dL, *p* < 0.001] and higher baseline Creatinine concentrations, coupled with a notable reduction in serum Albumin levels (*p* < 0.001). Consistently, the AKI group presented with a much higher global severity of illness, as indicated by elevated Sequential Organ Failure Assessment (SOFA) scores [median 7 vs. 4, *p* < 0.001]. Consequently, these high-risk individuals required a significantly greater intensity of critical care interventions, including higher Positive End-Expiratory Pressure (PEEP) support [median 5 vs. 0 cmH_2_O, *p* < 0.001], and an elevated frequency of vasopressor administration (83.1% vs. 59.8%, *p* < 0.001) and sedative/analgesic support (89.1% vs. 77.1%, *p* < 0.001).

### 3.2. Optimal Feature Selection via Machine Learning Intersections

To identify the most critical predictors of pneumothorax-associated AKI, a tri-algorithm machine learning intersection strategy—incorporating Boruta, Least Absolute Shrinkage and Selection Operator (LASSO) regression, and Recursive Feature Elimination (RFE)—was deployed. Detailed feature selection trajectories and coefficient shrinkage paths are provided in Supplementary Figure S1. By taking the precise intersection of these three distinct methodologies, a parsimonious core set of 7 critical variables was identified: Blood Urea Nitrogen (BUN), Sequential Organ Failure Assessment (SOFA) score, Chronic Kidney Disease (CKD), Positive End-Expiratory Pressure (PEEP), Heart Failure, Albumin, and Age.

### 3.3. Correlation and Clinical Landscape of Selected Predictors

**Figure 2:**
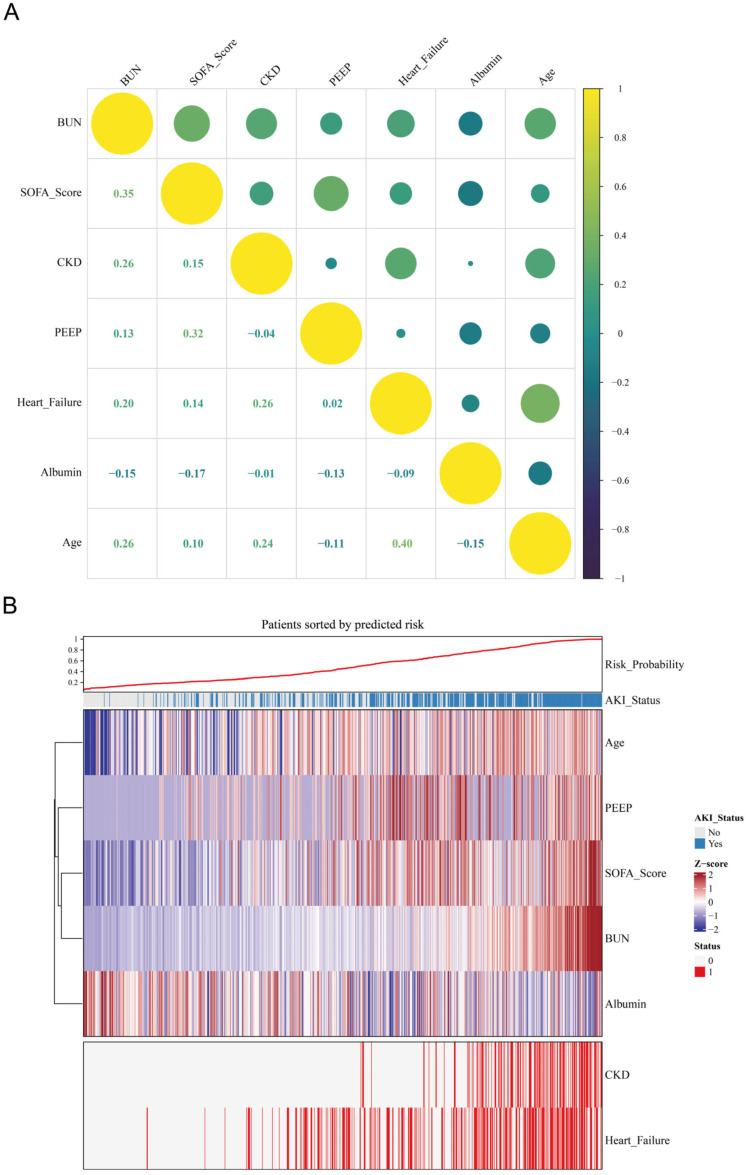
Clinical landscape and feature correlations of the selected predictors. (**A**) Spearman correlation matrix of the 7 core features. The upper right triangle visualizes correlation magnitudes using circle size and color intensity, while the lower left triangle presents the exact correlation coefficients. (**B**) Unsupervised hierarchical clustering and risk-sorted heatmap illustrating the distribution of continuous clinical characteristics (Z-score standardized) and categorical interventions across different AKI risk probabilities.

To decipher the clinical landscape and evaluate the multi-collinearity profile of the 7 locked core features, a Spearman correlation matrix and an unsupervised hierarchical clustering heatmap were constructed (Figure 2). The correlation matrix (Figure 2A) revealed weak-to-moderate statistical associations among the selected clinical parameters, confirming the absence of severe multicollinearity. Prominent positive associations were observed between Age and Heart Failure (r = 0.40), BUN and SOFA score (r = 0.35), as well as SOFA score and PEEP (r = 0.32), reflecting the intricate interplay between aging, illness severity, and aggressive respiratory interventions. Conversely, serum Albumin exhibited widespread negative correlations with severe indicators, such as BUN (r = −0.15) and SOFA score (r = −0.17).

The Z-score-normalized, risk-sorted clustering heatmap (Figure 2B) visually demonstrated distinct clinical phenotypes across the predicted risk gradient. As the predicted probability curve steadily escalated, patients who ultimately developed AKI exhibited visibly denser, high-expression clusters (red zones) for continuous variables including BUN, SOFA score, and PEEP, coupled with a high concentration of categorical risk factors (CKD and Heart Failure). This visualization validated the high discriminative potential of this 7-feature subset.

### 3.4. Algorithmic Comparison and Performance Evaluation

**Figure 3:**
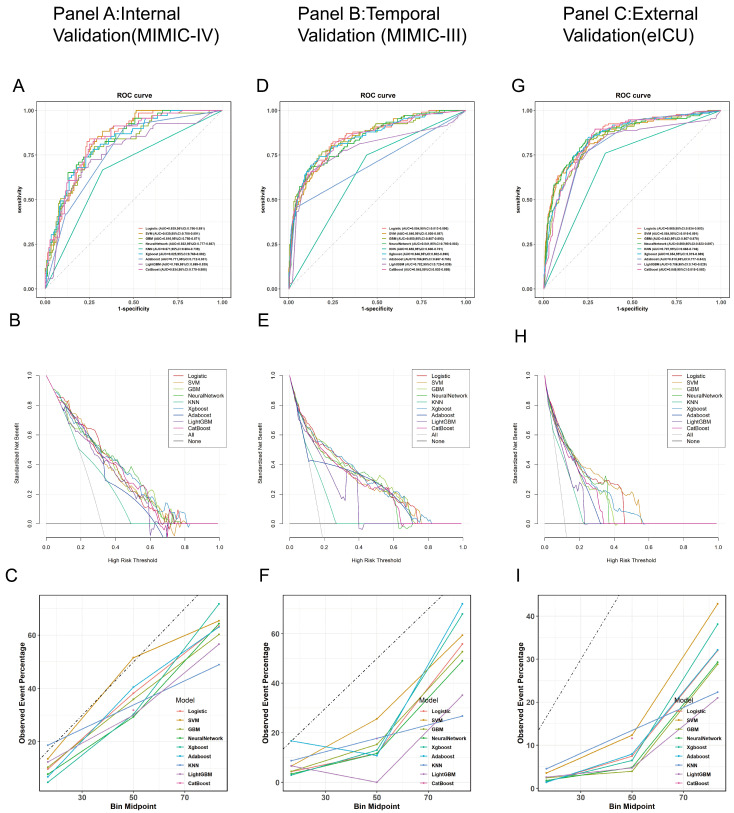
Comprehensive performance evaluation of the nine machine learning algorithms across three independent validation cohorts. The left column illustrates model discrimination (ROC) (**A**), clinical net benefit (DCA) (**B**), and risk calibration (**C**) in the internal validation cohort (MIMIC-IV). The middle column presents the corresponding ROC (**D**), DCA (**E**), and calibration curves (**F**) in the temporal validation cohort (MIMIC-III). The right column displays the ROC (**G**), DCA (**H**), and calibration curves (**I**) in the external eICU validation cohort. The diagonal dashed lines represent the reference line for random chance in the ROC curves and perfect agreement in the calibration curves. Pairwise AUC comparisons via DeLong’s test for all nine algorithms are provided in Supplementary Table S2.

The 7 core predictors were systematically fed into nine supervised machine learning algorithms to evaluate their discriminative capability, clinical utility, and calibration across all validation cohorts (Figure 3). The Logistic Regression model demonstrated consistent classification performance. In the internal validation cohort (MIMIC-IV), it achieved an Area Under the Curve (AUC) of 0.839 (95% CI: 0.786–0.891) [Sensitivity: 81.5%, Specificity: 76.0%, NPV: 95.0%, PPV: 42.1%], matching or exceeding complex ensemble methods like XGBoost (AUC = 0.825). This strong discriminative capacity and resistance to overfitting were confirmed in both the temporal validation cohort (MIMIC-III; AUC = 0.854) and the geographically distinct eICU external validation cohort (AUC = 0.869).

Regarding risk calibration, the Logistic Regression model showed acceptable alignment in the MIMIC-IV and MIMIC-III cohorts, though a noticeable calibration drift (Calibration Slope = 0.56) occurred in the heterogeneous eICU cohort, indicating a systematic tendency to overestimate the probability of AKI in high-risk patients and underestimate it in low-risk patients (detailed calibration metrics, including Brier scores and Hosmer-Lemeshow tests, are summarized in Supplementary Table S3). Decision Curve Analysis (DCA) confirmed that this parsimonious model yielded the highest standardized net clinical benefit across a wide range of threshold probabilities, performing comparably to the complex full-variable model (Supplementary Figure S2).

To further dissect predictive accuracy, confusion matrices across all validation tiers were generated (Supplementary Figure S3). The Logistic Regression model maintained a stable, balanced distribution of True Positive and True Negative rates, successfully minimizing the false-negative misclassification rate to safeguard timely clinical surveillance.

### 3.5. Feature Importance and Global Explanation

To ensure algorithmic transparency, feature importance scores across all nine models were evaluated (Supplementary Figure S4). Despite disparities in statistical architectures, BUN and the SOFA score were consistently identified as top-tier prognostic drivers, confirming that the 7-feature subset captures stable biological signals rather than dataset-specific noise.

**Figure 4:**
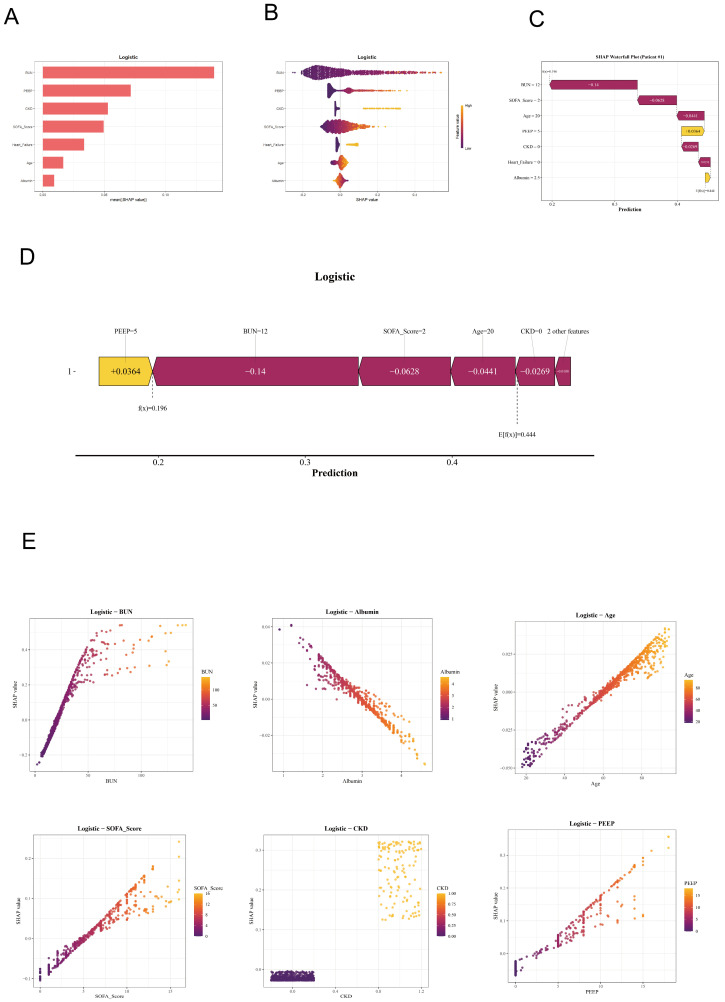
Multi-dimensional model interpretation using Shapley Additive exPlanations (SHAP) for the optimal regularized Logistic Regression engine. (**A**) Global SHAP summary bar plot based on mean absolute SHAP values; (**B**) SHAP beeswarm plot illustrating the directional risk impact of feature values (red indicating high values, blue indicating low values); (**C**) Local SHAP waterfall plot and (**D**) force plot demonstrating the individualized, step-by-step risk adjustment for a representative patient; (**E**) SHAP dependence scatter plots exploring the continuous risk trajectories and threshold inflection points for individual predictors. In the SHAP waterfall plot, purple arrows represent feature contributions that decrease the predicted risk of AKI, whereas yellow arrows represent contributions that increase the risk.

To demystify the internal decision-making mechanics of the optimal Logistic Regression model, a Shapley Additive exPlanations (SHAP) framework was implemented (Figure 4). Global SHAP analyses (Figure 4A,B) confirmed that elevated BUN, SOFA score, and PEEP, alongside pre-existing CKD and Heart Failure, strongly drove AKI prediction, whereas higher serum Albumin acted as a prominent protective factor. At the individual level (Figure 4C,D), local SHAP explanations dynamically adjusted the baseline risk probability based on specific patient parameters. SHAP dependence plots (Figure 4E) revealed distinct risk inflection thresholds—such as the SOFA score transitioning to positive risk at a threshold of 4—foreshadowing their complex non-linear dynamics.

### 3.6. Multivariate Regression and Non-Linear Dynamics

**Figure 5:**
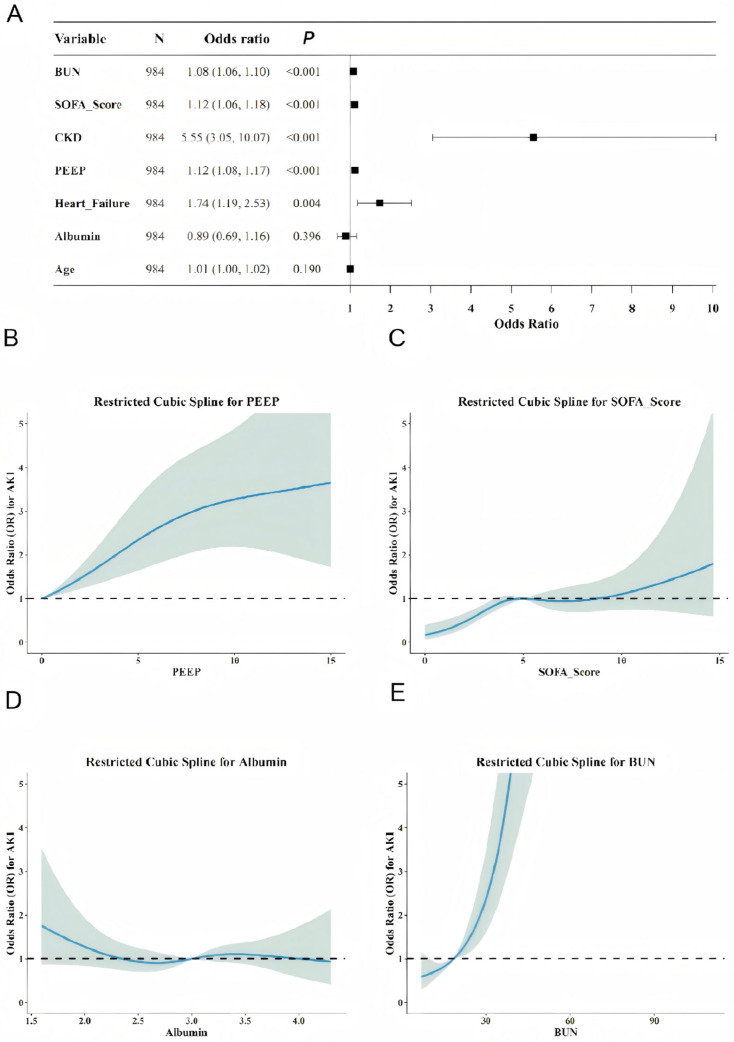
Traditional multivariable epidemiological validation and non-linear dose–response dynamics. (**A**) Forest plot illustrating the independent Odds Ratios (ORs), 95% confidence intervals (CIs), and corresponding *p*-values for the 7 core predictors within the fully adjusted multivariable logistic regression framework; (**B**–**E**) Restricted Cubic Spline (RCS) curves validating the adjusted non-linear risk trajectories for key continuous parameters: (**B**) Positive End-Expiratory Pressure (PEEP), (**C**) Sequential Organ Failure Assessment (SOFA) score, (**D**) serum Albumin, and (**E**) Blood Urea Nitrogen (BUN). The solid blue lines represent the estimated OR values, the shaded areas indicate the 95% CIs, and the horizontal dashed lines mark the reference boundary of OR = 1.0. For the Restricted Cubic Spline (RCS) plots, the solid blue lines represent the estimated odds ratios (OR), and the shaded areas denote the 95% confidence intervals (CI). The horizontal dashed lines indicate the reference line of OR = 1.

To evaluate the independent prognostic value of the selected variables, a multivariable logistic regression analysis was performed, and the results were visualized using an odds ratio (OR) forest plot, as shown in Figure 5A. The multivariable analysis demonstrated that pre-existing Chronic Kidney Disease (CKD) was the strongest independent predictor of pneumothorax-associated AKI, exhibiting a significant risk elevation (OR = 5.55, 95% CI: 3.05–10.07, *p* < 0.001). Elevated baseline severity markers also presented significant independent hazardous effects, including the SOFA score (OR = 1.12, 95% CI: 1.06–1.18, *p* < 0.001) and Blood Urea Nitrogen (BUN) (OR = 1.08, 95% CI: 1.06–1.10, *p* < 0.001). Positive airway pressure therapy was identified as a notable clinical risk driver, with each unit increase in Positive End-Expiratory Pressure (PEEP) loads driving a 12% escalation in AKI probability (OR = 1.12, 95% CI: 1.08–1.17, *p* < 0.001). Pre-existing Heart Failure also retained statistical significance (OR = 1.74, 95% CI: 1.19–2.53, *p* = 0.004). Conversely, serum Albumin (OR = 0.89, *p* = 0.396) and Age (OR = 1.01, *p* = 0.190) did not demonstrate independent linear significance within the fully adjusted multivariable matrix.

To capture the potential complex mathematical constraints that traditional linear constraints often obscure, Restricted Cubic Spline (RCS) analyses were generated to granularly model the continuous risk trajectories, as illustrated in Figure 5B–E. The dose–response curve for PEEP (Figure 5B) demonstrated a continuous, progressive risk acceleration that plateaued at higher operational loads, validating that elevated intrathoracic pressures consistently compromise renal perfusion. For the baseline SOFA score (Figure 5C), a distinct biphasic trajectory was discovered; the risk of AKI escalated sharply from a score of 0 up to a critical inflection threshold of approximately 5, after which the curve demonstrated a brief stabilization before maintaining a gradual upward gradient under severe organ dysfunction cascades.

Continuous metabolic and nutritional markers demonstrated non-linear configurations. The adjusted risk trajectory for serum Albumin (Figure 5D) assumed a distinct non-linear configuration, characterized by a steep escalation in AKI probability within the severe hypoalbuminemia domain (<2.5 g/dL), which gradually flattened and reached a safe nadir as concentrations approached normal ranges. The risk curve for BUN (Figure 5E) manifested an exponential risk acceleration. While initially remaining flat within the normal physiological spectrum, the individual probability of AKI experienced a sharp risk escalation once BUN values crossed the clinical inflection threshold of approximately 20–25 mg/dL, highlighting its potential utility as an early biochemical risk marker for acute renal decompensation.

### 3.7. Subgroup Consistency and Robustness Check

**Figure 6:**
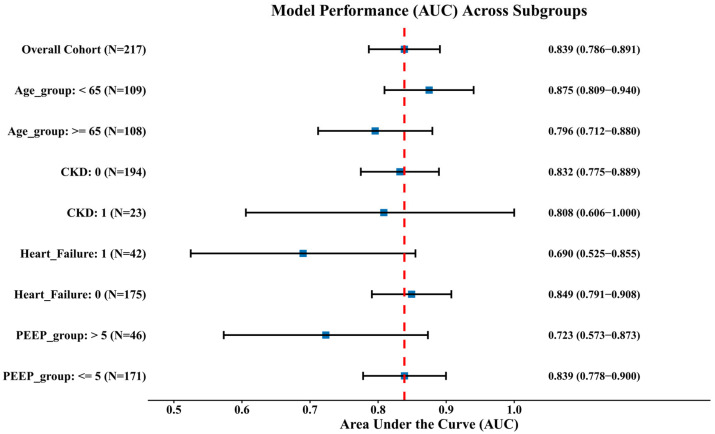
Multivariable subgroup analysis validating the predictive consistency and robustness of the regularized Logistic Regression model. The forest plot displays the precise Area Under the Curve (AUC) metrics alongside corresponding 95% confidence intervals (CIs) calculated across distinct age groups, chronic kidney disease (CKD) statuses, preexisting heart failure conditions, and positive end-expiratory pressure (PEEP) treatment thresholds within the validation cohort. The dashed vertical red line indicates the baseline diagnostic benchmark of the overall cohort (0.839). The blue squares represent the point estimates of the Area Under the Curve (AUC), and the horizontal black lines represent the corresponding 95% confidence intervals. The vertical red dashed line indicates the baseline diagnostic benchmark of the overall cohort.

To rigorously verify the stability and clinical robustness of the optimal regularized Logistic Regression model across heterogeneous clinical phenotypes, a comprehensive subgroup analysis was conducted within the validation cohort (N = 217), as illustrated in Figure 6. The model achieved an overall discriminative efficacy, yielding a baseline Area Under the Curve (AUC) of 0.839 (95% CI: 0.786–0.891).

When stratified by major clinical demographics and baseline confounding parameters, the predictive engine demonstrated performance stability. In the age-based stratifications, the model exhibited stable classification accuracy among younger individuals (<65 years, N = 109) with an reliable AUC of 0.875 (95% CI: 0.809–0.940). Even when confronted with the high-risk geriatric sub-population (≥65 years, N = 108), whose complex multi-organ interactions often obscure predictive pathways, the algorithm maintained an acceptable and stable predictive performance with an AUC of 0.796 (95% CI: 0.712–0.880).

The model’s generalizability was examined across distinct comorbidity backgrounds and ventilatory therapeutic boundaries. For patients partitioned by baseline renal impairment, the algorithm yielded consistent predictive performance between non-CKD individuals (N = 194, AUC = 0.832) and those with pre-existing CKD (N = 23, AUC = 0.808), demonstrating high resistance to baseline renal noise. In terms of respiratory therapeutics, the predictive framework retained an identical AUC of 0.839 (95% CI: 0.778–0.900) within the standard low Positive End-Expiratory Pressure sub-population (PEEP ≤ 5 cmH_2_O, N = 171). Although a marginal reduction in performance boundaries was observed in complicated clinical subsets—specifically patients undergoing aggressive positive airway pressure therapy (PEEP > 5 cmH_2_O, AUC = 0.723) and those with concurrent acute-on-chronic cardiac decompensation (Heart Failure = 1, AUC = 0.690)—the lower bounds of their 95% CIs consistently remained above the random classification threshold (0.50), supporting the consistency of the developed risk stratification tool.

### 3.8. Clinical Deployment: Static and Dynamic Tools

**Figure 7:**
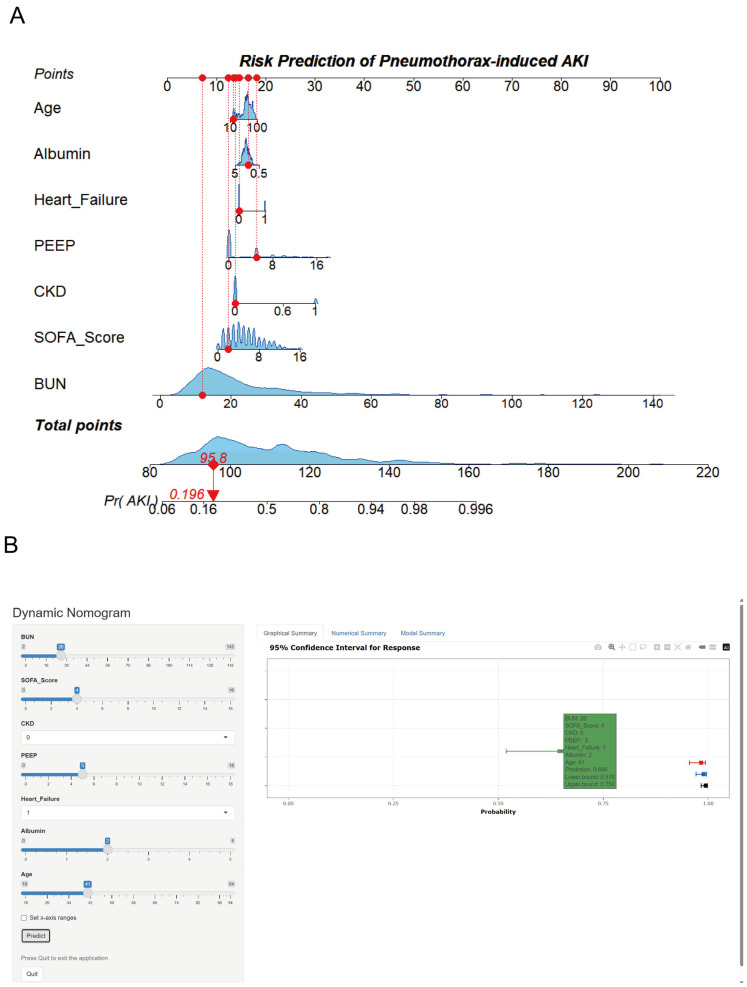
Dual clinical deployment channels for the optimal risk stratification framework. (**A**) Advanced, density-integrated static nomogram incorporating population distribution profiles across the continuous predictor axes for manual risk estimation; In Panel (**A**), blue areas represent density distributions, and red dots with lines indicate specific values for an individual patient. (**B**) Operational preview of the dynamic, web-based interactive prediction calculator developed using the R Shiny framework, demonstrating its real-time digital computation and individualized visual explanation capabilities.

To translate the regularized Logistic Regression framework into pragmatic, user-friendly clinical workflows at the bedside, both static and dynamic visual decision-making tools were successfully constructed, as illustrated in Figure 7.

First, a static, density-enhanced alignment nomogram was developed to provide a rapid, non-computational mathematical calculation system for clinicians (Figure 7A). Unlike traditional, minimalist nomograms that conceal the underlying data distribution, this advanced iteration integrates high-resolution probability density plots within each continuous parameter axis (BUN, SOFA score, PEEP, and Albumin). This addition allows clinicians to simultaneously evaluate an individual patient’s absolute variable measurement alongside their relative standing within the overall critical care cohort. By mapping a patient’s specific clinical profile upward to the “Points” axis, summing the derived segments to determine the “Total Points,” and projecting that sum downward, medical staff can visually estimate an individual’s precise mathematical probability of developing pneumothorax-associated AKI within seconds.

Second, to overcome the visual interpolation limits of static paper charts and support digital intensive care units (eICUs), the predictive engine was developed into a dynamic, web-based interactive calculation app via the R Shiny framework (freely accessible at: https://guanghao.shinyapps.io/AKI_Predictor/) (accessed on 5 July 2026). A representative operational interface of this digital portal is shown in Figure 7B. Through this secure graphical user interface, clinicians can input a patient’s seven core parameters via intuitive sliders and drop-down menus. The remote cloud engine executes the regularized mathematical equation in real time, immediately rendering an exact risk percentage alongside interactive, individualized SHAP breakdown graphs. This dual-deployment strategy effectively bridges the gap between complex machine learning and bedside precision medicine, serving primarily as a rule-out instrument. Given its high Negative Predictive Value (NPV = 95.0%) and moderate Positive Predictive Value (PPV = 42.1%), this tool excels at identifying low-risk patients to safely avoid excessive interventions. As indicated by the external calibration drift, local recalibration is explicitly required before this web calculator can be used at any institution outside the development cohort.

## 4. Discussion

The primary objective of this multicenter retrospective study was to develop, validate, and interpret a machine learning framework for predicting ICU-acquired Acute Kidney Injury (AKI) in patients presenting with pneumothorax. By executing atri-algorithm intersection strategy (Boruta, LASSO, and RFE), we identified a parsimonious core subset of 7 clinically accessible predictors from a high-dimensional dataset. During the comprehensive algorithmic evaluation, the regularized Logistic Regression model demonstrated consistent discriminative power, achieving high AUCs in the internal MIMIC-IV cohort (0.839) and across the temporal validation cohort (MIMIC-III, AUC = 0.854) and independent external validation cohort (eICU, AUC = 0.869). By integrating SHAP and Restricted Cubic Spline (RCS) analyses, we transcended the traditional algorithmic “black-box” paradigm to unveil critical non-linear pathophysiological dynamics. The final deployment of a density-enhanced nomogram and a dynamic web-based calculator translates this complex mathematical architecture into an actionable, real-time bedside triage tool. Recent advancements in intensive care data science further corroborate that interpretable machine learning frameworks significantly outperform conventional scoring systems in predicting dynamic AKI trajectories [22,23,24].

The methodological strength of our study lies in the feature engineering and the unexpected, yet rational, improved performance of the linear framework. Traditional predictive modeling in critical care is frequently compromised by subjective variable selection and the overfitting tendencies of single algorithms [25]. Our tri-algorithm pipeline inherently mitigated multi-collinearity and dataset-specific noise. Interestingly, the Logistic Regression model effectively matched or outperformed advanced ensemble trees (e.g., XGBoost and LightGBM) and neural networks, particularly in external validation. This phenomenon aligns with emerging clinical data science consensus: for structured, tabular medical data with predefined baseline features, appropriately regularized linear models often exhibit superior geographical generalizability by penalizing extreme, over-fitted decision boundaries that complex tree algorithms tend to exploit [26].

Pathophysiologically, the specific predictors identified by our framework capture the complex hemodynamic and neurohormonal cascades linked to AKI in pneumothorax patients. Global SHAP analysis identified BUN, SOFA score, and PEEP as top-tier risk drivers. Severe pneumothorax intrinsically elevates intrathoracic pressure; when aggressive mechanical ventilation (high PEEP) is superimposed, this mechanical shift severely impedes systemic venous return. This not only precipitates a drop in cardiac output and arterial underfilling [27] but also induces venous congestion. Elevated central venous pressure directly translates to increased renal venous pressure, critically reducing the net glomerular filtration gradient [7]. This hypoperfusion state triggers essential neurohormonal compensatory mechanisms. The activation of the Renin–Angiotensin–Aldosterone System (RAAS) and sympathetic nervous stimulation independently drive severe renal vasoconstriction, exacerbating ischemic injury beyond pure mechanical compression [28,29]. This mechanical and neurohormonal cascade is further exacerbated in patients with pre-existing Heart Failure, whose compromised cardiac reserves render them highly susceptible to acute preload reductions [28].

In our multivariable framework, pre-existing CKD emerged as the strongest independent predictor (OR = 5.55). This prominent finding reflects the diminished renal functional reserve in these patients, rendering their microvasculature vulnerable to the aforementioned hemodynamic fluctuations and neurohormonal insults. Compared to general ICU AKI predictive models [22,23,24], our pneumothorax-specific framework incorporates the unique mechanical constraints (e.g., PEEP loads) intrinsic to this vulnerable population. This specific clinical contextualization allows our parsimonious model to maintain competitive discriminative accuracy (AUC 0.839–0.869) despite a smaller, homogeneous cohort, highlighting the prognostic value of disease-specific feature extraction over broad, generalized ICU data mining.

Beyond linear associations, our RCS analyses unveiled critical non-linear thresholds that traditional clinical epidemiology often overlooks. The risk curve for BUN demonstrated an exponential escalation once values crossed a physiological threshold of approximately 20–25 mg/dL. While serum creatinine remains the gold standard for defining AKI, its rise is often delayed [29]. BUN, conversely, is responsive to early neurohormonal activation and urea reabsorption triggered by transient renal hypoperfusion [30]. Thus, the exponential risk acceleration of BUN identified in our model serves as a biochemical risk marker for impending acute renal decompensation. Similarly, the protective role of serum Albumin underscores the impact of systemic inflammation and capillary leak syndrome; severe hypoalbuminemia accelerates intravascular volume depletion, further compromising the delicate renal microcirculation [31]. It is noteworthy that although Age and serum Albumin did not reach conventional statistical significance (*p* > 0.05) in the standard multivariable logistic regression, they were deliberately retained in the final framework. Unlike traditional linear models that rely strictly on isolated *p*-values, our ML feature selection pipeline (LASSO and RFE) evaluates holistic predictive contributions and complex non-linear interactions. From a clinical perspective, advanced age is strongly associated with diminished baseline renal functional reserve [18], and hypoalbuminemia critically exacerbates ischemic vulnerability [31]. Retaining these universally recognized variables ensures the model’s clinical face validity and preserves its generalizability in real-world intensive care settings.

Regarding clinical implementation, the predictive values of our model require careful contextualization. While the framework achieves a high Negative Predictive Value (NPV) of 95.0%, its Positive Predictive Value (PPV) stands at 42.1%. This implies that fewer than half of the patients flagged as high-risk will actually develop AKI. Therefore, this tool is intended to serve primarily as a “rule-out” instrument rather than a definitive “rule-in” diagnostic test. Clinicians can leverage the high NPV to confidently identify low-risk patients, thereby safely avoiding unnecessary and resource-intensive interventions. Conversely, a high-risk prediction should not dictate immediate aggressive treatments; rather, it should prompt heightened bedside vigilance, closer monitoring of urine output, and proactive optimization of modifiable factors, such as careful PEEP titration.

Despite the encouraging discriminative performance and clinical net benefit validated via DCA, several limitations of this study must be acknowledged. First, inherent to its retrospective observational design across the MIMIC and eICU databases, unmeasured confounders—such as the exact radiological volume of the pneumothorax, the presence of tension pneumothorax, or the precise timing of chest tube thoracostomy—could not be fully incorporated. Second, our algorithm exclusively utilizes static data extracted within the first 24 h of ICU admission. Given the dynamic nature of critical illness, incorporating continuous longitudinal trajectories of respiratory and renal biomarkers might further optimize predictive accuracy [32]. Future prospective, multicenter clinical trials incorporating dynamic physiological waveforms and multi-omics data are warranted to refine and validate our findings prior to routine clinical adoption. Third, some subgroup analyses in this study involved relatively small sample sizes (e.g., specific comorbidity strata), which may lead to limited statistical power and wide confidence intervals; thus, these exploratory findings should be interpreted with caution. Fourth, this retrospective secondary analysis using publicly available EHR databases was not prospectively registered on PROSPERO or other platforms before data extraction, which may introduce analytical flexibility bias. Prospective registration will be adopted in our future prospective validation research. While the model maintained robust discrimination, calibration drift (Calibration Slope = 0.56) was observed in the heterogeneous eICU multicenter database. This shrinkage naturally reflects the diverse clinical practices, varying intensive care thresholds, and differing baseline AKI incidences across hundreds of distinct institutions. This finding transparently highlights the universal challenge of model transportability and underscores a critical clinical consensus: local recalibration is a necessary prerequisite before deploying single-center derived algorithms into completely distinct geographical or multi-center healthcare systems. While the overall occurrence of AKI was rigorously defined according to guidelines, the granular distribution of KDIGO stages (Stages 1–3) among the positive cases could not be reliably reported. This limitation is inherent to the retrospective nature of the extracted clinical dataset, which predominantly captured the binary diagnostic event of AKI rather than the continuous, dynamic trajectories of daily serum creatinine and urine output required for precise sub-staging classification.

In conclusion, we successfully developed and externally validated a transparent, data-driven machine learning framework to predict pneumothorax-associated AKI. By elucidating complex non-linear clinical dynamics and deploying accessible digital tools, this study equips intensive care physicians with a prognostic instrument to optimize proactive bedside interventions, individualize mechanical ventilation strategies, and ultimately support clinical decision-making.

## 5. Conclusions

In conclusion, this study successfully developed and externally validated a transparent, parsimonious machine learning framework for the early prediction of Acute Kidney Injury (AKI) in critically ill patients with pneumothorax. By utilizing a rigorous tri-algorithm intersection strategy, we identified a core subset of seven clinically accessible predictors: Blood Urea Nitrogen (BUN), the SOFA score, Chronic Kidney Disease (CKD), Positive End-Expiratory Pressure (PEEP), Heart Failure, Albumin, and Age. Among the competitive algorithms, the regularized Logistic Regression model demonstrated consistent discriminative performance, acceptable mathematical calibration, and acceptable clinical utility, which were consistently replicated across the internal MIMIC-IV database and validated using a temporal validation cohort (MIMIC-III) and an independent external validation cohort (eICU).

The integration of explainable artificial intelligence (XAI) workflows, specifically SHAP and Restricted Cubic Spline (RCS) analyses, demystified the algorithmic “black box,” unveiling important non-linear dose–response trajectories and clinical risk thresholds. The successful multi-channel deployment of the optimal model as an advanced static density-enhanced nomogram and an interactive web-based calculation tool bridges the gap between complex computational science and bedside medical practice. Ultimately, this data-driven early warning system offers a potential prognostic instrument to assist in personalized risk stratification, refine mechanical ventilation strategies, and facilitate timely therapeutic interventions and assist in risk assessment. Nevertheless, large-scale prospective validation is required before this framework can be adopted into routine clinical practice.

## Figures and Tables

**Table 1:** Baseline Demographic and Clinical Characteristics of the Study Population.

Variables	Total (*n* = 1040)	AKI = 0 (*n* = 702)	AKI = 1 (*n* = 338)	*p*-Value
CKD, *n* (%)				
0	937 (90.1%)	672 (95.7%)	265 (78.4%)	<0.001
1	103 (9.9%)	30 (4.3%)	73 (21.6%)	
COPD, *n* (%)				
0	868 (83.5%)	602 (85.8%)	266 (78.7%)	0.005
1	172 (16.5%)	100 (14.2%)	72 (21.3%)	
Heart_Failure, *n* (%)				
0	828 (79.6%)	608 (86.6%)	220 (65.1%)	<0.001
1	212 (20.4%)	94 (13.4%)	118 (34.9%)	
Sedatives_Analgesics, *n* (%)				
0	198 (19.0%)	161 (22.9%)	37 (10.9%)	<0.001
1	842 (81.0%)	541 (77.1%)	301 (89.1%)	
Sepsis, *n* (%)				
0	389 (37.4%)	312 (44.4%)	77 (22.8%)	<0.001
1	651 (62.6%)	390 (55.6%)	261 (77.2%)	
Vasopressor, *n* (%)				
0	339 (32.6%)	282 (40.2%)	57 (16.9%)	<0.001
1	701 (67.4%)	420 (59.8%)	281 (83.1%)	
Age, median [IQR]	64.0 [49.0–74.0]	62.0 [43.0–71.0]	67.0 [57.0–79.0]	<0.001
Albumin, median [IQR]	3.0 [2.5–3.4]	3.0 [2.6–3.4]	2.8 [2.3–3.2]	<0.001
BUN, median [IQR]	17.0 [12.0–26.0]	15.0 [11.0–20.0]	26.5 [18.0–42.0]	<0.001
Creatinine, median [IQR]	0.9 [0.7–1.2]	0.8 [0.6–1.0]	1.3 [0.9–1.9]	<0.001
PEEP, median [IQR]	0.0 [0.0–5.0]	0.0 [0.0–5.0]	5.0 [0.0–8.0]	<0.001
SOFA_Score, median [IQR]	5.0 [2.0–7.0]	4.0 [2.0–6.0]	7.0 [4.0–10.0]	<0.001

## Data Availability

The data that support the findings of this study are available from the MIMIC-IV (https://physionet.org/content/mimiciv/ (accessed on 5 July 2026)), MIMIC-III (https://physionet.org/content/mimiciii/ (accessed on 5 July 2026)), and eICU (https://physionet.org/content/eicu-crd/ (accessed on 5 July 2026)) databases upon approved request and successful completion of the required credentialing via the PhysioNet platform. The complete R source code and the underlying R Shiny application code (R version 4.3.1) used for data extraction, preprocessing, model development, and statistical analysis presented in this study are publicly available. The code can be accessed permanently on Zenodo (DOI: 10.5281/zenodo.20777551) and on GitHub (https://github.com/ThoracicSurgeon/AKI_Predictor_Shiny (accessed on 5 July 2026)).

## Supplementary Materials

The following supporting information can be downloaded at https://www.mdpi.com/article/10.3390/jcm15145599/s1, Figure S1: Optimal feature selection via a tri-algorithm machine learning intersection strategy; Figure S2: Decision Curve Analysis (DCA) of the 7-variable model versus the full-variable model; Figure S3: Granular comparison of predictive performance via multi-panel confusion matrices; Figure S4: Standardized feature importance scores across the nine competitive machine learning algorithms; Table S1: Comprehensive baseline characteristics of the development cohort; Table S2: Pairwise AUC comparisons via DeLong’s test; Table S3: Calibration metrics across validation cohorts; File S1: Completed TRIPOD + AI checklist; File S2: Raw R analysis codes for data extraction, model construction, and Shiny calculator development.

## Abbreviations

The following abbreviations are used in this manuscript:

Abbreviation	Full Term
AKI	Acute Kidney Injury
AUC	Area Under the Curve
BUN	Blood Urea Nitrogen
CI	Confidence Interval
CKD	Chronic Kidney Disease
DCA	Decision Curve Analysis
eICU	eICU Collaborative Research Database
GBM	Gradient Boosting Machine
ICU	Intensive Care Unit
KNNs	K-Nearest Neighbors
LASSO	Least Absolute Shrinkage and Selection Operator
MIMIC	Medical Information Mart for Intensive Care
OR	Odds Ratio
PEEP	Positive End-Expiratory Pressure
RCS	Restricted Cubic Spline
RFE	Recursive Feature Elimination
ROC	Receiver Operating Characteristic
SHAP	Shapley Additive exPlanations
SOFA	Sequential Organ Failure Assessment
SVM	Support Vector Machine
XAI	Explainable Artificial Intelligence

## Appendix A. Mathematical Formulation of the Predictive Model

### Appendix A.1. Logistic Regression Equation

The static nomogram and the dynamic web-based calculator developed in this study are fundamentally associated with the optimized multivariable logistic regression equation. The probability of pneumothorax-associated acute kidney injury (AKI), denoted as $P$, is calculated using the following logistic function:

P = 1/(1 + e^(−L)^)

where L (the linear predictor) is defined by the model’s intercept and the sum of the products of the feature values and their corresponding coefficients (β):

L = β0 + β1 × 1 + β2 × 2 + β3 × 3 + … + β7 × 7

### Appendix A.2. Specific Predictive Equation for Pneumothorax-Associated AKI

L = −2.957 + (0.075 × BUN) + (0.109 × SOFA_Score) + (1.713 × CKD[Yes = 1, No = 0]) + (0.117 × PEEP) + (0.551 × Heart_Failure[Yes = 1, No = 0]) − (0.114 × Albumin) + (0.006 × Age)

Note: For binary categorical variables such as CKD and Heart Failure, a value of 1 is assigned if the condition is present (“Yes”), and 0 if it is absent (“No”). Continuous variables such as BUN, SOFA score, PEEP, Albumin, and Age are inputted with their exact numerical values.
