# Supplementary material for: Development and External Validation of an Interpretable Machine Learning Framework for Predicting Pneumothorax-Associated Acute Kidney Injury: A Multicenter Retrospective Study
Source: J Clin Med. 2026 Jul 16;15(14):5599. doi: 10.3390/jcm15145599 (PMC13412805; doi:10.3390/jcm15145599)
Supplement: Supplementary file 1 [file jcm-15-05599-s001.zip › jcm-4352079-supplementary.pdf]

Supplementary Figure S1

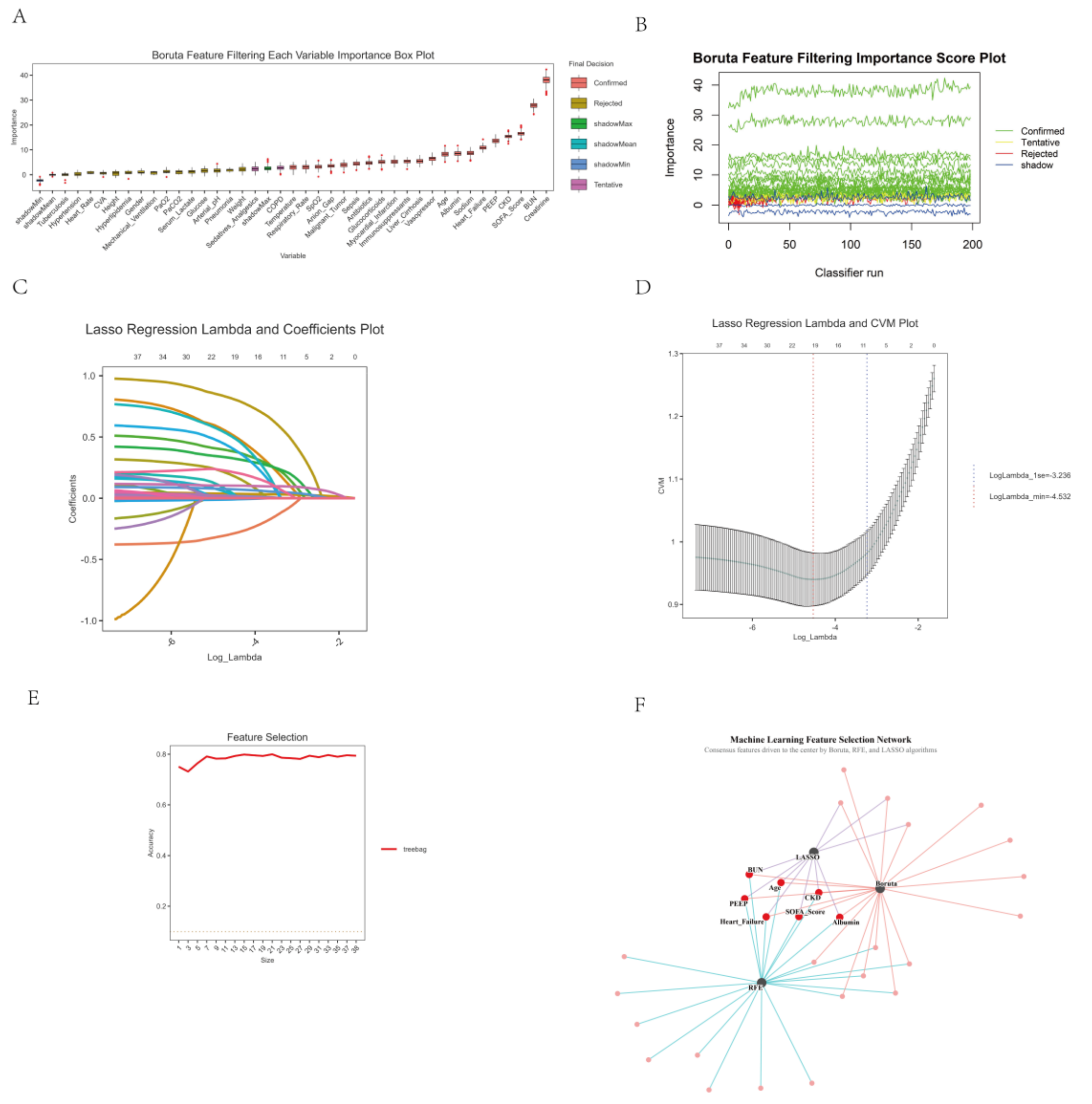

Supplementary Figure S1

Optimal feature selection via a tri-algorithm machine learning intersection strategy. (A,B) Boruta algorithm feature filtering, displaying variable importance box plots and score trajectories to identify confirmed relevant predictors. (C,D) Least Absolute Shrinkage and Selection Operator (LASSO) regression analysis, illustrating coefficient shrinkage paths and the selection of the optimal penalty parameter (λ) via 10-fold cross-validation. (E) Recursive Feature Elimination (RFE) process demonstrating the relationship between the number of selected features and model accuracy. (F) A feature selection network diagram illustrating the strict intersection of the three distinct algorithms. The core variables uniformly identified by Boruta, LASSO, and RFE are driven to the center, yielding a parsimonious subset of 7 optimal predictors (BUN, Age, CKD, SOFA Score, Albumin, Heart Failure, and PEEP).

Supplementary Figure S2

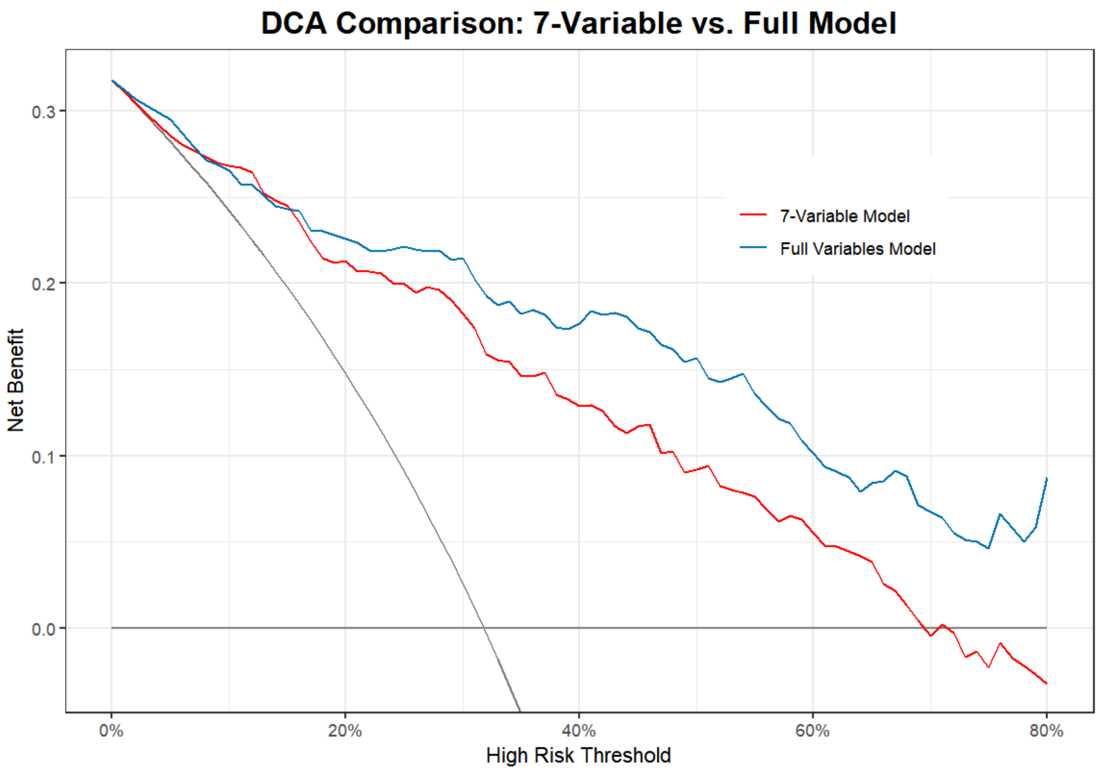

Supplementary Figure S2 Decision Curve Analysis (DCA) of the 7-variable model versus the full-variable model. The red line represents the parsimonious 7-variable model, while the blue line represents the complex full-variable model. The gray solid line indicates the "treat-all" strategy, and the diagonal gray line indicates the "treat-none" strategy. The y-axis represents the standardized net clinical benefit across a wide range of high-risk threshold probabilities, demonstrating that the 7-variable model provides comparable clinical utility to the full-variable model.

Supplementary Figure S3

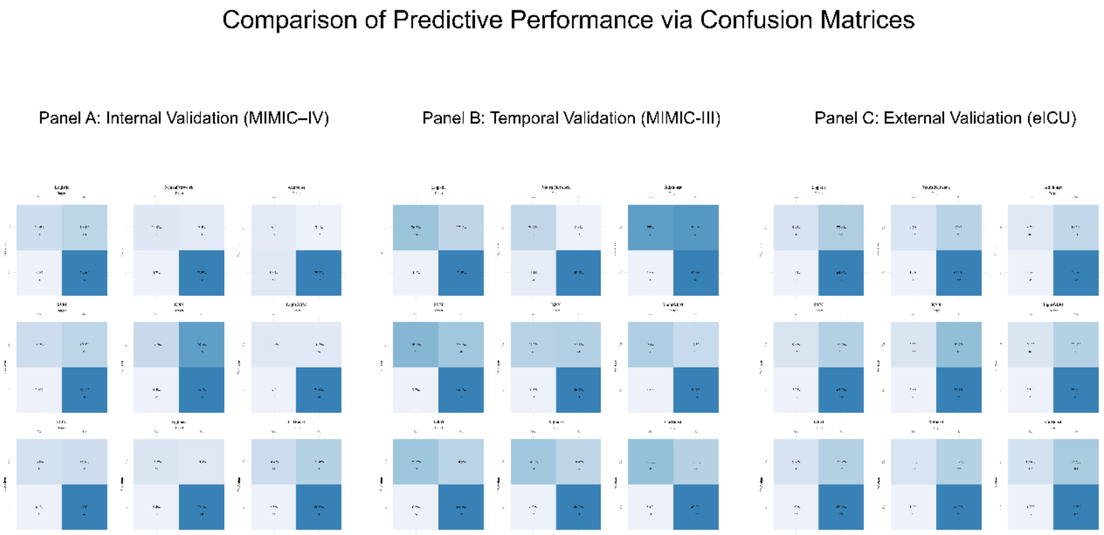

Supplementary Figure S3 Granular comparison of predictive performance via multi-panel confusion matrices. The matrices detail the exact classification counts and percentage rates for all nine competitive machine learning models within the internal validation cohort (Panel A), temporal validation cohort (MIMIC-III) (Panel B), and independent external validation cohort (eICU) (Panel C), highlights the superior and balanced performance of the regularized Logistic Regression engine.

Supplementary Figure S4

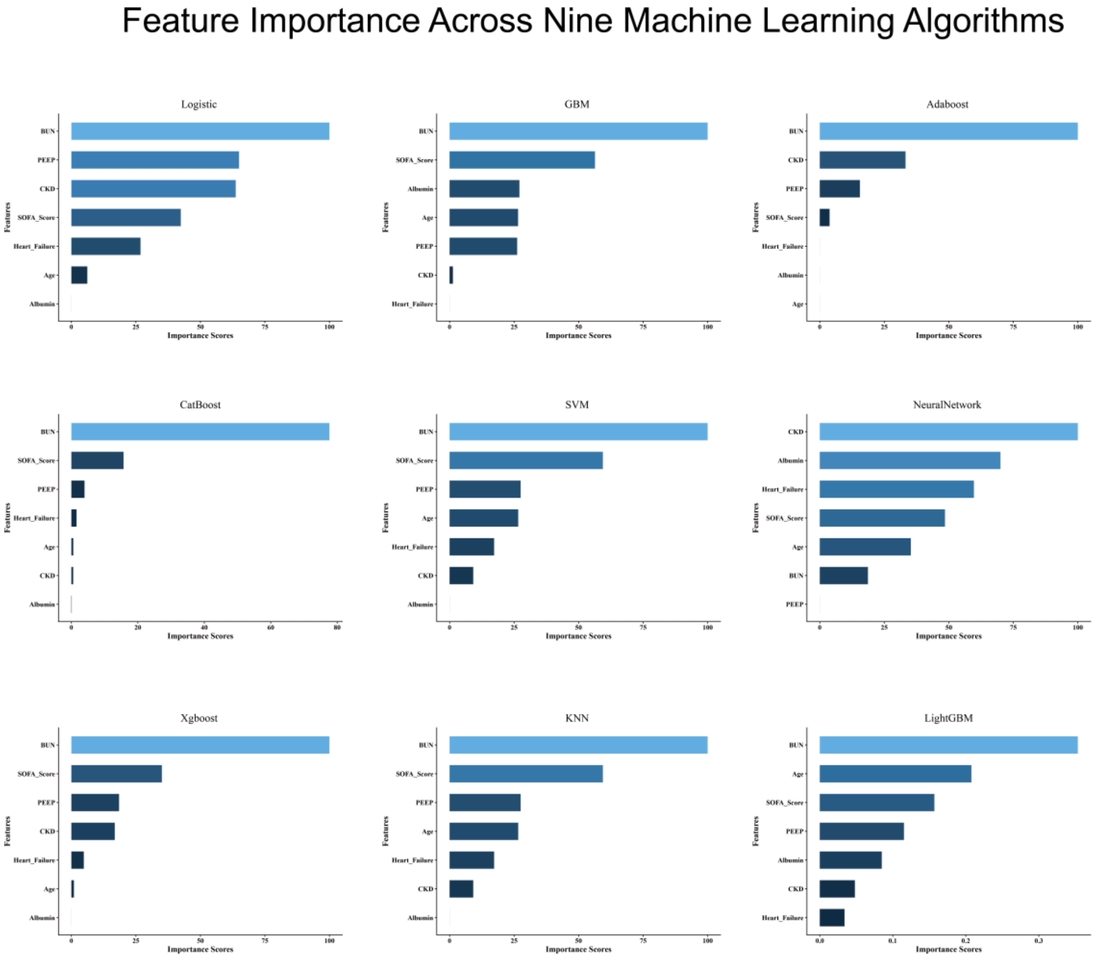

Supplementary Figure S4 Standardized feature importance scores across the nine competitive machine learning algorithms. The bar charts demonstrate the cross-algorithmic consensus, confirming that Blood Urea Nitrogen (BUN) and Sequential Organ Failure Assessment (SOFA) score consistently serve as the primary prognostic drivers regardless of mathematical architecture.

Supplementary Table S1

Variables	Total (n=1040)	AKI=0 (n=702)	AKI=1 (n=338)	P-value
Antibiotics, n (%)				
0	144 (13.8%)	131 (18.7%)	13 (3.8%)	<0.001
1	896 (86.2%)	571 (81.3%)	325 (96.2%)	
CKD, n (%)				
0	937 (90.1%)	672 (95.7%)	265 (78.4%)	<0.001
1	103 (9.9%)	30 (4.3%)	73 (21.6%)	
COPD, n (%)				
0	868 (83.5%)	602 (85.8%)	266 (78.7%)	0.005
1	172 (16.5%)	100 (14.2%)	72 (21.3%)	
CVA, n (%)				
0	981 (94.3%)	664 (94.6%)	317 (93.8%)	0.705
1	59 (5.7%)	38 (5.4%)	21 (6.2%)	
Gender, n (%)				
F	393 (37.8%)	259 (36.9%)	134 (39.6%)	0.43
M	647 (62.2%)	443 (63.1%)	204 (60.4%)	
Glucocorticoids, n (%)				
0	785 (75.5%)	570 (81.2%)	215 (63.6%)	<0.001
1	255 (24.5%)	132 (18.8%)	123 (36.4%)	
Heart_Failure, n (%)				
0	828 (79.6%)	608 (86.6%)	220 (65.1%)	<0.001
1	212 (20.4%)	94 (13.4%)	118 (34.9%)	
Hyperlipidemia, n (%)				
0	743 (71.4%)	512 (72.9%)	231 (68.3%)	0.144
1	297 (28.6%)	190 (27.1%)	107 (31.7%)	

Hypertension, n (%)				
0	669 (64.3%)	441 (62.8%)	228 (67.5%)	0.164
1	371 (35.7%)	261 (37.2%)	110 (32.5%)	
Immunosuppressants, n (%)				
0	1015 (97.6%)	693 (98.7%)	322 (95.3%)	0.001
1	25 (2.4%)	9 (1.3%)	16 (4.7%)	
Liver_Cirrhosis, n (%)				
0	990 (95.2%)	684 (97.4%)	306 (90.5%)	<0.001
1	50 (4.8%)	18 (2.6%)	32 (9.5%)	
Malignant_Tumor, n (%)				
0	951 (91.4%)	650 (92.6%)	301 (89.1%)	0.073
1	89 (8.6%)	52 (7.4%)	37 (10.9%)	
Mechanical_Ventilation, n (%)				
0	77 (7.4%)	59 (8.4%)	18 (5.3%)	0.099
1	963 (92.6%)	643 (91.6%)	320 (94.7%)	
Myocardial_Infarction, n (%)				
0	986 (94.8%)	680 (96.9%)	306 (90.5%)	<0.001
1	54 (5.2%)	22 (3.1%)	32 (9.5%)	
Pneumonia, n (%)				
0	684 (65.8%)	499 (71.1%)	185 (54.7%)	<0.001
1	356 (34.2%)	203 (28.9%)	153 (45.3%)	
Sedatives_Analgesics, n (%)				
0	198 (19.0%)	161 (22.9%)	37 (10.9%)	<0.001
1	842 (81.0%)	541 (77.1%)	301 (89.1%)	

Sepsis, n (%)				
0	389 (37.4%)	312 (44.4%)	77 (22.8%)	<0.001
1	651 (62.6%)	390 (55.6%)	261 (77.2%)	
Tuberculosis, n (%)				
0	1015 (97.6%)	686 (97.7%)	329 (97.3%)	0.871
1	25 (2.4%)	16 (2.3%)	9 (2.7%)	
Vasopressor, n (%)				
0	339 (32.6%)	282 (40.2%)	57 (16.9%)	<0.001
1	701 (67.4%)	420 (59.8%)	281 (83.1%)	
Age, median [IQR]	64.0 [49.0-74.0]	62.0 [43.0-71.0]	67.0 [57.0-79.0]	<0.001
Albumin, median [IQR]	3.0 [2.5-3.4]	3.0 [2.6-3.4]	2.8 [2.3-3.2]	<0.001
Anion_Gap, median [IQR]	14.0 [11.0-16.0]	13.0 [11.0-16.0]	15.0 [12.0-18.0]	<0.001
Arterial_pH, median [IQR]	7.4 [7.3-7.4]	7.4 [7.3-7.4]	7.3 [7.3-7.4]	0.004
BUN, median [IQR]	17.0 [12.0-26.0]	15.0 [11.0-20.0]	26.5 [18.0-42.0]	<0.001
Bicarbonate, median [IQR]	23.0 [20.0-25.0]	23.0 [21.0-25.0]	22.0 [19.0-25.0]	<0.001
Creatinine, median [IQR]	0.9 [0.7-1.2]	0.8 [0.6-1.0]	1.3 [0.9-1.9]	<0.001
Glucose, median [IQR]	7.4 [6.1-9.2]	7.2 [6.1-8.8]	7.8 [6.2-10.1]	0.004
Heart_Rate, median [IQR]	90.0 [79.0-104.0]	90.0 [79.0-103.0]	91.0 [78.0-107.0]	0.255
Height, median [IQR]	170.0 [163.0-178.0]	170.0 [163.0-178.0]	170.0 [163.0-178.0]	0.162
PEEP, median [IQR]	0.0 [0.0-5.0]	0.0 [0.0-5.0]	5.0 [0.0-8.0]	<0.001
PaCO2, median [IQR]	43.0 [38.0-51.0]	43.0 [38.0-50.8]	43.0 [37.0-51.0]	0.623

PaO2, median [IQR]	116.0 [70.0-236.0]	122.0 [74.0-254.0]	101.0 [65.0-204.0]	0.004
Respiratory_Rate, median [IQR]	19.0 [15.0-24.0]	18.0 [15.0-23.0]	20.0 [16.0-25.0]	<0.001
SOFA_Score, median [IQR]	5.0 [2.0-7.0]	4.0 [2.0-6.0]	7.0 [4.0-10.0]	<0.001
Serum_Lactate, median [IQR]	1.8 [1.2-2.8]	1.8 [1.2-2.7]	1.9 [1.2-3.0]	0.069
Sodium, median [IQR]	139.0 [136.0-141.0]	139.0 [136.0-141.0]	139.0 [135.0-142.0]	0.939
SpO2, median [IQR]	98.0 [95.0-100.0]	99.0 [96.0-100.0]	98.0 [94.0-100.0]	<0.001
Temperature, median [IQR]	36.9 [36.6-37.2]	36.9 [36.7-37.2]	36.8 [36.5-37.2]	0.007
Weight, median [IQR]	76.8 [64.0-91.1]	76.0 [63.5-90.0]	77.3 [64.3-93.9]	0.189

Supplementary Table S2

**Reference Model**	**Compared Model**	**AUC (Reference)**	**AUC (Compared)**	**Z value**	**P value**
Logistic Regression	SVM	0.854	0.846	0.964	0.335
Logistic Regression	GBM	0.854	0.850	0.310	0.756
Logistic Regression	Neural Network	0.854	0.841	1.181	0.238
Logistic Regression	KNN	0.854	0.655	7.767	< 0.001
Logistic	XGBoost	0.854	0.846	1.023	0.306

Reference Model	Compared Model	AUC (Reference)	AUC (Compared)	Z value	P value
Regression					
Logistic Regression	AdaBoost	0.854	0.706	6.811	< 0.001
Logistic Regression	LightGBM	0.854	0.782	3.350	< 0.001
Logistic Regression	CatBoost	0.854	0.845	0.902	0.367

Supplementary Table S3

Cohort	Calibration Intercept	Calibration Slope	Brier Score	Hosmer-Lemeshow P value
**Training (MIMIC-IV)**	0.000	1.000	0.148	0.320
**Internal Validation (MIMIC-IV)**	-0.456	0.683	0.160	< 0.001
**Temporal External Validation (MIMIC-III)**	-1.176	0.905	0.120	< 0.001

Cohort	Calibration Intercept	Calibration Slope	Brier Score	Hosmer-Lemeshow P value
**Spatial External Validation (eICU)**	-2.246	0.565	0.154	< 0.001
