# Supplementary material for: Development and External Validation of an Interpretable Machine Learning Framework for Predicting Pneumothorax-Associated Acute Kidney Injury: A Multicenter Retrospective Study
Source: J Clin Med. 2026 Jul 16;15(14):5599. doi: 10.3390/jcm15145599 (PMC13412805; doi:10.3390/jcm15145599)
Supplement: Supplementary file 1 [file jcm-15-05599-s001.zip › jcm-4352079-supplementary1.pdf]

File S1: TRIPOD+AI Checklist

TRIPOD+AI Checklist

Complete Reporting of Prediction Models Based on Machine Learning and Artificial Intelligence

Section/Topic	Item	Checklist item	Reported on Section/Page
**TITLE**			
Title	1	Identify the study as developing or evaluating the performance of a multivariable prediction model, the target population, and the outcome to be predicted	**Title page**
**ABSTRACT**			
Abstract	2	See TRIPOD+AI for Abstracts checklist	**Abstract**
**INTRODUCTION**			
Background / Objectives	3a	Explain the healthcare context and rationale for developing or evaluating the prediction model, including references to existing models	**1. Introduction**
	3b	Describe the target population and the intended purpose of the prediction model in the context of the care pathway, including its intended users (e.g., healthcare professionals, patients, public)	**1. Introduction**
	3c	Describe any known health inequalities between sociodemographic groups	N/A (Not explicitly addressed)
	4	Specify the study objectives, including whether the study describes the development or validation of a prediction model (or both)	**1. Introduction**
**METHODS**			
Data	5a	Describe the sources of data separately for the development and evaluation datasets (e.g., randomised trial, cohort, routine care or registry data), the rationale for using these data, and representativeness of the data	**2.1. Study Design and Ethical Considerations**
	5b	Specify the dates of the collected participant data, including start and end of participant accrual; and, if applicable, end of follow-up	2.2. Study Population and Data
Participants	6a	Specify key elements of the study setting (e.g., primary care, secondary care, general population) including the number and location of centres	**2.1. Study Design and Ethical Considerations**
	6b	Describe the eligibility criteria for study participants	**2.2. Study Population and Data**
	6c	Give details of any treatments received, and how they were handled during model development or evaluation, if relevant	**2.2. Study Population and Data & Table 1**
Data preparation	7	Describe any data pre-processing and quality checking, including whether this was similar across relevant sociodemographic groups	**2.2. Study Population and Data**
Outcome	8a	Clearly define the outcome that is being predicted and the time horizon, including how and when assessed, the rationale for choosing this outcome, and whether the method of outcome assessment is consistent across sociodemographic groups	**2.2. Study Population and Data**
	8b	If outcome assessment requires subjective interpretation, describe the qualifications and demographic characteristics of the outcome assessors	N/A (Objective EHR definition)
	8c	Report any actions to blind assessment of the outcome to be predicted	N/A (Retrospective study)
Predictors	9a	Describe the choice of initial predictors (e.g., literature, previous models, all available predictors) and any pre-selection of predictors before model building	**2.3. Optimal Feature Selection Strategy**
	9b	Clearly define all predictors, including how and when they were measured (and any actions to blind assessment of predictors for the outcome and other predictors)	**2.2. Study Population and Data**

	9c	If predictor measurement requires subjective interpretation, describe the qualifications and demographic characteristics of the predictor assessors	N/A (Objective EHR variables)
Sample size	10	Explain how the study size was arrived at (separately for development and evaluation), and justify that the study size was sufficient to answer the research question. Include details of any sample size calculation	**2.2. Study Population (Numbers provided)**
Missing data	11	Describe how missing data were handled. Provide reasons for omitting any data	**2.2. Study Population and Data**
Analytical methods	12a	Describe how the data were used (e.g., for development and evaluation of model performance) in the analysis, including whether the data were partitioned, considering any sample size requirements	**2.4. Model Development and Comprehensive Evaluation**
	12b	Depending on the type of model, describe how predictors were handled in the analyses (functional form, rescaling, transformation, or any standardisation)	**2.4. Model Development and Comprehensive Evaluation**
	12c	Specify the type of model, rationale, all model-building steps, including any hyperparameter tuning, and method for internal validation	**2.4. Model Development and Comprehensive Evaluation**
	12d	Describe if and how any heterogeneity in estimates of model parameter values and model performance was handled and quantified across clusters (e.g., hospitals, countries)	**2.4. Model Development and Comprehensive Evaluation**
	12e	Specify all measures and plots used (and their rationale) to evaluate model performance (e.g., discrimination, calibration, clinical utility) and, if relevant, to compare multiple models	**2.4. Model Development and Comprehensive Evaluation**
	12f	Describe any model updating (e.g., recalibration) arising from the model evaluation, either overall or for particular sociodemographic groups or settings	**4. Discussion**
	12g	For model evaluation, describe how the model predictions were calculated (e.g., formula, code, object, application programming interface)	**2.5. Model Interpretation and Clinical Translation & Appendix A**
Class imbalance	13	If class imbalance methods were used, state why and how this was done, and any subsequent methods to recalibrate the model or the model predictions	**2.4. Model Development and Comprehensive Evaluation**
Fairness	14	Describe any approaches that were used to address model fairness and their rationale	Not reported
Model output	15	Specify the output of the prediction model (e.g., probabilities, classification). Provide details and rationale for any classification and how the thresholds were identified	**2.5. Model Interpretation and Clinical Translation & Appendix A**
Training vs evaluation	16	Identify any differences between the development and evaluation data in healthcare setting, eligibility criteria, outcome, and predictors	**2.1 & 2.2**
Ethical approval	17	Name the institutional research board or ethics committee that approved the study and describe the participant-informed consent or the ethics committee waiver of informed consent	**Institutional Review Board Statement**
**OPEN SCIENCE**			
Funding	18a	Give the source of funding and the role of the funders for the present study	Funding
Conflicts of interest	18b	Declare any conflicts of interest and financial disclosures for all authors	**Conflicts of Interest**
Protocol / Registration	18c, 18d	Indicate where the study protocol can be accessed or state that a protocol was not prepared / Provide registration information for the study, including register name and registration number	4. Discussion (Future prospective registration mentioned)
Data sharing / Code sharing	18e, 18f	Provide details of the availability of the study data / Provide details of the availability of the analytical code	**Data Availability Statement**
**PATIENT & PUBLIC INVOLVEMENT**			
Patient & Public Involvement	19	Provide details of any patient and public involvement during the design, conduct, reporting, interpretation, or dissemination of the study or state no involvement	Not reported
**RESULTS**			
Participants	20a	Describe the flow of participants through the study, including the number of participants with and without the outcome and, if applicable, a summary of the follow-up time. A diagram may be helpful	**3.1. Baseline Characteristics of the Study Population & Figure 1 & Table 1**
	20b	Report the characteristics overall and, where applicable, for each data source or setting, including the key dates, key predictors (including demographics), treatments received, sample size, number of outcome events, follow-up time, and amount of missing data. A table may be helpful	**3.1. Baseline Characteristics of the Study Population & Figure 1 & Table 1**
	20c	For model evaluation, show a comparison with the development data of the distribution of important predictors (demographics, predictors, and outcome)	**3.1 & Supplementary Table S1**
Model specification	21	Specify the number of participants and outcome events in each analysis (e.g., for model development, hyperparameter tuning, model evaluation)	**4.1. Baseline Characteristics**

	22	Provide details of the full prediction model (e.g., formula, code, object, application programming interface) to allow predictions in new individuals and to enable third-party evaluation and implementation, including any restrictions to access or re-use	**3.8. Clinical Deployment: Static and Dynamic Tools & Appendix A**
Model performance	23a	Report model performance estimates with confidence intervals, including for any key subgroups (e.g., sociodemographic). Consider plots to aid presentation	**3.4. Algorithmic Comparison and Performance Evaluation & Figure 3**
	23b	If examined, report results of any heterogeneity in model performance across clusters. See TRIPOD-Cluster for additional details	**3.7. Subgroup Consistency and Robustness Check & Figure 6**
Model updating	24	Report the results from any model updating, including the updated model and subsequent performance	**4.3 & Supp Table S3**
**DISCUSSION**			
Interpretation	25	Give an overall interpretation of the main results, including issues of fairness in the context of the objectives and previous studies	**4. Discussion**
Limitations	26	Discuss any limitations of the study (such as a non-representative sample, sample size, overfitting, missing data) and their effects on any biases, statistical uncertainty, and generalizability	**4. Discussion**
Usability	27a	Describe how poor quality or unavailable input data (e.g., predictor values) should be assessed and handled when implementing the prediction model	Not reported
	27b	Specify whether users will be required to interact in the handling of the input data or use of the model, and what level of expertise is required of users	**3.8. Clinical Deployment**
	27c	Discuss any next steps for future research, with a specific view to applicability and generalizability of the model	**4. Discussion & 5. Conclusions**

D = items relevant only to development of a prediction model; E = items relating solely to evaluation of a prediction model; D;E = items applicable to both.

From: Collins GS, Moons KGM, Dhiman P, et al. BMJ 2024;385:e078378. doi:10.1136/bmj-2023-078378

Version: 11-January-2024

File S2: Raw R analysis codes

```
# ==========================================================================
# Script Name: R_analysis_code.R
# Description: Machine Learning Pipeline for Predicting Pneumothorax-Associated
#              Acute Kidney Injury (AKI) in the Intensive Care Unit.
# Authors: Guanghao Pan, et al.
# Institution: First Hospital of Hebei Medical University
```

```
#
# Structure:
#   Part 1: Environment Setup & Data Preprocessing (SMOTE)
#   Part 2: Machine Learning Model Training & Hyperparameter Tuning
#   Part 3: Model Evaluation (ROC, Calibration, DCA, Confusion Matrix)
#   Part 4: Interpretability (SHAP Values & Visualization)
#   Part 5: Clinical Application Tools (RCS, Forest Plot, Nomogram)
# ==============================================================================

# ==============================================================================
# PART 1: Environment Setup & Data Preprocessing
# ==============================================================================
# Load required libraries (Comprehensive list for data processing, ML, and plotting)
library(caret)
library(pROC)
library(ggplot2)
library(rms)
library(rmda)
library(dcurves)
library(DMwR)       # For SMOTE
library(catboost)
```

```
library(lightgbm)
library(kernelshap)
library(shapviz)
library(patchwork)
library(cvms)
library(tibble)
library(dplyr)
library(forestplot)

# Set Working Directory (Users should modify this path to their local environment)
# setwd("path_to_your_project_folder")

# Define target variable and core predictors identified from prior analyses
core_vars <- c("AKI", "BUN", "SOFA_Score", "CKD", "PEEP", "Heart_Failure", "Albumin", "Age")

# Load Development Data (MIMIC-IV) and Format
data <- read.csv("data.csv", header = TRUE)
data$AKI <- factor(data$AKI, levels = c(0, 1), labels = c('No', 'Yes'))

# Data Partitioning (70% Training, 30% Internal Validation)
set.seed(999)
```

```
inTrain <- createDataPartition(y = data$AKI, p = 0.7, list = FALSE)

traindata <- data[inTrain, core_vars]

testdata  <- data[-inTrain, core_vars]


# Load Validation Data (Temporal: MIMIC-III / External: eICU)

validdata <- read.csv("valid.csv", header = TRUE)

validdata$AKI <- factor(validdata$AKI, levels = c(0, 1), labels = c('No', 'Yes'))

extra <- validdata[, core_vars]


# Handle Class Imbalance using SMOTE on Training Data

set.seed(123)

newData <- SMOTE(AKI ~ ., data = traindata, k = 5, perc.over = 203, perc.under = 150)

dev <- newData

vad <- testdata

dev$AKI <- factor(as.character(dev$AKI))


# ============================================================================

# PART 2: Machine Learning Model Training & Hyperparameter Tuning

# ============================================================================

# Define models to be trained using caret package

models <- c("glm", "svmRadial", "gbm", "nnet", "xgbTree", "AdaBoost.M1", "kknn")
```

```
models_names <- list(Logistic="glm", SVM="svmRadial", GBM="gbm", NeuralNetwork="nnet",
                     Xgboost="xgbTree", Adaboost="AdaBoost.M1", KNN="kknn")

# Hyperparameter Tuning Grids (Optimized to prevent overfitting)
Tune_table <- list(
  glm         = NULL,
  svmRadial   = expand.grid(sigma = 0.001, C = 0.09),
  gbm         = expand.grid(n.trees = 110, interaction.depth = 5, shrinkage = 0.1, n.minobsinnode = 30),
  nnet        = expand.grid(size = 4, decay = 0.4),
  kknn        = expand.grid(kmax = 12, distance = 1, kernel = "optimal"),
  xgbTree     = expand.grid(nrounds = 150, max_depth = 2, eta = 0.01, gamma = 10,
                            colsample_bytree = 0.5, min_child_weight = 5, subsample = 0.6),
  AdaBoost.M1 = expand.grid(mfinal = 2, maxdepth = 2, coeflearn = "Zhu")
)

# Initialize storage for predictions and models
train_probe <- data.frame(AKI = dev$AKI)
test_probe  <- data.frame(AKI = vad$AKI)
extra_probe <- data.frame(AKI = extra$AKI)
ML_calss_model <- list()
importance <- list()
```

```
# Train standard models using 10-fold cross-validation
train.control <- trainControl(method = 'repeatedcv', number = 10, repeats = 5,
                              classProbs = TRUE, summaryFunction = twoClassSummary)

cat("Training Caret Models...\n")
for (model in models) {
  model_name <- names(models_names)[which(models_names == model)]
  set.seed(52)
  fit <- train(AKI ~ ., data = dev, tuneGrid = Tune_table[[model]], metric = 'ROC',
               method = model, trControl = train.control)

  train_probe[[model_name]] <- predict(fit, newdata = dev, type = 'prob')$Yes
  test_probe[[model_name]]  <- predict(fit, newdata = vad, type = 'prob')$Yes
  extra_probe[[model_name]] <- predict(fit, newdata = extra, type = 'prob')$Yes

  ML_calss_model[[model_name]] <- fit
  importance[[model_name]]     <- varImp(fit, scale = TRUE)
}

# --- Train Specialized Tree-based Models (LightGBM & CatBoost) ---
```

```r
cat("Training LightGBM & CatBoost...\n")

# LightGBM Setup
dtrain <- lgb.Dataset(as.matrix(dev[, -1]), label = ifelse(dev$AKI=="Yes", 1, 0))
lgb_params <- list(objective = "binary", metric = "auc", min_data = 1L, learning_rate = 1.0)
lgb_model <- lgb.train(params = lgb_params, data = dtrain, nrounds = 5L)

train_probe$LightGBM <- predict(lgb_model, as.matrix(dev[, -1]))
test_probe$LightGBM  <- predict(lgb_model, as.matrix(vad[, -1]))
extra_probe$LightGBM <- predict(lgb_model, as.matrix(extra[, -1]))
ML_calss_model$LightGBM <- lgb_model

# CatBoost Setup (with restricted parameters to avoid overfitting)
train_pool <- catboost.load_pool(as.matrix(dev[, -1]), label = ifelse(dev$AKI=="Yes", 1, 0))
test_pool  <- catboost.load_pool(as.matrix(vad[, -1]), label = ifelse(vad$AKI=="Yes", 1, 0))
cat_params <- list(iterations = 80, learning_rate = 0.005, depth = 2,
                   l2_leaf_reg = 50, eval_metric = 'AUC', logging_level = 'Silent')

cat_model <- catboost.train(train_pool, test_pool, cat_params)
train_probe$CatBoost <- catboost.predict(cat_model, train_pool, prediction_type = 'Probability')
test_probe$CatBoost  <- catboost.predict(cat_model, test_pool, prediction_type = 'Probability')
```

```
ML_calss_model$CatBoost <- cat_model


# ==============================================================================
# PART 3: Model Evaluation (Calibration, ROC, DCA, Confusion Matrix)
# ==============================================================================
# (Representative code for plotting internal validation cohort metrics)


# ROC Curve Generation
ROC_list <- list()
for (m_name in c(names(models_names), "LightGBM", "CatBoost")) {
  ROC_list[[m_name]] <- roc(response = test_probe$AKI, predictor = test_probe[, m_name])
}
ROC_plot <- ggroc(ROC_list, size = 1.2, legacy.axes = TRUE) + theme_bw() +
  labs(title = 'ROC Curves (Internal Validation)') +
  geom_segment(aes(x = 0, y = 0, xend = 1, yend = 1), colour = 'grey', linetype = 'dashed')
ggsave("ROC_Internal_Validation.pdf", ROC_plot, width = 7, height = 7)


# Decision Curve Analysis (DCA)
dca_formula <- as.formula("AKI ~ Logistic")
dca_test <- decision_curve(dca_formula, data = test_probe %>% mutate(AKI = ifelse(AKI=="Yes",1,0)),
                           study.design = "cohort", bootstraps = 50)
```

```r
pdf("DCA_Logistic_Test.pdf", width = 6, height = 6)

plot_decision_curve(dca_test, curve.names = "Logistic Nomogram")

dev.off()


# High-resolution Calibration Curve (Logistic)

cal_test_data <- vad %>% mutate(AKI = ifelse(AKI=="Yes",1,0))

dd <- datadist(cal_test_data); options(datadist = "dd")

fit_lrm <- lrm(AKI ~ SOFA_Score + BUN + PEEP + Albumin + Age + CKD + Heart_Failure,

               data = cal_test_data, x = TRUE, y = TRUE)

pred_vad <- predict(fit_lrm, type = "fitted")


pdf("Calibration_Single_Test.pdf", width = 6, height = 6)

val.prob(pred_vad, cal_test_data$AKI, pl = TRUE, cex = 0.8, xlab = "Predicted Probability")

title("Calibration Curve (Internal Validation)")

dev.off()


# ============================================================================

# PART 4: Interpretability (SHAP Values & Visualization)

# ============================================================================

# Utilizing KernelSHAP for model-agnostic explanation (Example using Logistic)

best_Model <- "Logistic"
```

```
clean_dev <- na.omit(dev)

p_fun <- function(model, newdata) {
  res <- predict(model, newdata = as.data.frame(newdata), type = "prob", na.action = na.pass)[["Yes"]]
  res[is.na(res)] <- 0
  return(res)
}

explain_kernel <- kernelshap(
  object = ML_calss_model[[best_Model]],
  X = clean_dev[1:min(966, nrow(clean_dev)), -1],
  bg_X = clean_dev[1:100, -1],
  pred_fun = p_fun
)

shp_viz <- shapviz(explain_kernel)

# SHAP Beeswarm Plot
pdf(paste0("SHAP_", best_Model, "_Beeswarm.pdf"), width = 8, height = 6)
sv_importance(shp_viz, kind = "beeswarm", max_display = 15) + theme_bw() +
  scale_color_gradient(low = "#1a85ff", high = "#d41159")
```

```r
dev.off()

# ==============================================================================
# PART 5: Clinical Application Tools (RCS, Forest Plot, Nomogram)
# ==============================================================================

# 1. Nomogram
pdf("Nomogram_AKI.pdf", width = 11, height = 8)
nom <- nomogram(fit_lrm, fun = function(x) 1/(1+exp(-x)),
                fun.at = c(0.1, 0.3, 0.5, 0.7, 0.9), funlabel = "Risk of AKI", lp = FALSE)
plot(nom)
dev.off()

# 2. Restricted Cubic Spline (RCS) - Example for BUN
fit_rcs <- lrm(AKI ~ rcs(BUN, 4) + SOFA_Score + PEEP + Albumin + Age + CKD + Heart_Failure,
               data = cal_test_data)
pred_rcs <- Predict(fit_rcs, BUN, fun = exp, ref.zero = TRUE)

pdf("RCS_BUN.pdf", width = 6, height = 5)
ggplot(as.data.frame(pred_rcs)) +
  geom_ribbon(aes(x = BUN, ymin = lower, ymax = upper), fill = "#84B0A5", alpha = 0.4) +
```

```r
  geom_line(aes(x = BUN, y = yhat), color = "#3690C0", linewidth = 1.2) +
  geom_hline(yintercept = 1, linetype = "dashed", color = "black") +
  theme_classic() + coord_cartesian(ylim = c(0, 5)) +
  labs(y = "Odds Ratio (OR)", title = "Dose-Response Association of BUN")
dev.off()

# 3. Dynamic Shiny App (DynNom)
# Uncomment the following lines to generate the web-based calculator
# library(DynNom)
# DynNom(fit_lrm, cal_test_data)

# --- END OF SCRIPT ---
```
